# Comparison of the new flexible tip bougie catheter and standard bougie stylet for tracheal intubation by anesthesiologists in different difficult airway scenarios: a randomized crossover trial

**DOI:** 10.1186/s12871-020-01009-7

**Published:** 2020-04-20

**Authors:** Kurt Ruetzler, Jacek Smereka, Cristian Abelairas-Gomez, Michael Frass, Marek Dabrowski, Szymon Bialka, Hanna Misiolek, Tadeusz Plusa, Oliver Robak, Olga Aniolek, Jerzy Robert Ladny, Damian Gorczyca, Sanchit Ahuja, Lukasz Szarpak

**Affiliations:** 1 Departments of Outcomes Research and General Anesthesia, Cleveland Clinic, Anesthesiology Institute, Cleveland, OH USA; 2 Department of Emergency Medical Service, Wroclaw Medical University, Wroclaw, Poland; 3 CLINURSID Research Group, University of Santiago de Compostela, Santiago de Compostela, Spain; 4 Faculty of Education, University Santiago de Compostela, Santiago de Compostela, Spain; 5 Institute of Research of Santiago (IDIS) and SAMID-II Network, Santiago de Compostela, Spain; 6 Department of Internal Medicine I, Medical University of Vienna, Vienna, Austria; 7 Chair and Department of Medical Education, Poznan University of Medical Sciences, Poznan, Poland; 8 Department of Anaesthesiology and Critical Care, School of Medicine with Division of Dentistry in Zabrze, Medical University of Silesia, Zabrze, Poland; 9 Medical Faculty, Lazarski University, Warsaw, Poland; 10 Polish Society of Disaster Medicine, Swieradowska 43 Str, 02-662 Warsaw, Poland; 11 Department of Emergency Medicine, Medical University Bialystok, Bialystok, Poland; 12 Department of Anesthesia, Henry Ford Health System, Detroit, MI USA

**Keywords:** Airway management, Endotracheal intubation, Medical simulation, Bougie catheter

## Abstract

**Background:**

Incidence of difficult endotracheal intubation ranges between 3 and 10%. Bougies have been recommended as an airway adjunct for difficult intubation, but reported success rates are variable. A new generation flexible tip bougie appears promising but was not investigated so far. We therefore compared the new flexible tip with a standard bougie in simulated normal and difficult airway scenarios, and used by experienced anesthesiologists.

**Methods:**

We conducted a observational, randomized, cross-over simulation study. Following standardized training, experienced anesthesiologists performed endotracheal intubation using a Macintosh blade and one of the bougies in six different airway scenarios in a randomized sequence: normal airway, tongue edema, pharyngeal obstruction, manual cervical inline stabilization, cervical collar stabilization, cervical collar stabilization and pharyngeal obstruction**.** Overall success rate with a maximum of 3 intubation attempts was the primary endpoint. Secondary endpoints included number of intubation attempts, time to intubation and dental compression.

**Results:**

Thirty-two anesthesiologist participated in this study between January 2019 and May 2019. Overall success rate was similar for the flexible tip bougie and the standard bougie. The flexible tip bougie tended to need less intubation attempts in more difficult airway scenarios. Time to intubation was less if using the flexible tip bougie compared to the standard bougie. Reduced severity of dental compression was noted for the flexible tip bougie in difficult airway scenarios except cervical collar stabilization.

**Conclusion:**

In this simulation study of normal and difficult airways scenarios, overall success rate was similar for the flexible tip and standard bougie. Especially in more difficult airway scenarios, less intubation attempts, and less optimization maneuvers were needed if using the flexible tip bougie.

**Trial registration:**

clinicaltrials.gov Identifier: NCT03733158. 7th November 2018.

## Background

During induction of anesthesia, the estimated incidence of difficult endotracheal intubation ranges between 3 and 10%, depending on the definition used [1, 2]. Recent advances in airways adjuncts like the introduction of videolaryngoscopes into clinical practice have led to fewer life-threatening complications, however the risk of serious complications still remains. Despite protracted convalescent, the current definitions to predict difficult airway situations are inadequate and often times prove unchallenging [3, 4]. Conversely, unanticipated difficult airway scenarios occur when least expected and significantly lead to anesthesia-related morbidity. The majority of these scenarios arise due to poor visualization of laryngeal inlet - “epiglottis only view” ostensibly due to condition such as pharyngeal obstruction, obesity, limited cervical mobility etc. [5–7]. Situations in which glottic view is expected to improve by external laryngeal manipulation — a readily available airway adjunct device (commonly known as bougie) is recommended to assist tracheal intubation.

A recent study in the emergency care setting demonstrated, that the use of a bougie resulted in a higher first attempt success rate when compared to conventional endotracheal intubation [8]. Previous work also reported the utility of bougie in difficult airway scenarios (such as cervical spine injuries) with a reported success rate ranging between 74 to 99% [9–12]. The variable success rate of the standard bougie was most commonly attributed to the inability to insert the bougie through the hypopharynx and laryngeal inlet [13]. To overcome this limitation, a new generation flexible tip bougie is designed to flexibly navigate the distal tip and help facilitate precise insertion of the endotracheal tube — even in a hyper curve airway [14]. The flexible tip bougie has an integrated slider along the surface which moves the tip anterior and posterior while the pre-curved distal portion of shaft allows the angulation to provide anterior flexion. The flexible tip is held, inserted and used like a standard bougie, except the intubator has an additional ability to navigate the bougie tip.

Intuitively the new flexible tip bougie seems to be a valuable device but the efficacy has not been investigated in the difficult airway setting yet. We therefore conducted a randomized cross over study to evaluate the usefulness of this new device, and used by experienced anesthesiologists in several airway manikin scenarios. We hypothesized that the new flexible tip bougie would perform comparably to the standard bougie in the normal airway scenario. In the difficult airway (tongue edema, manual in-line stabilization, or cervical collar stabilization), we hypothesized that the new flexible tip bougie would prove superior to the standard bougie.

## Methods

### Study design

This was an observational, randomized, cross-over simulation study. The study protocol was approved by the Institutional Review Board (IRB) of the Polish Society of Disaster Medicine (Approval no: 21.11.2017.IRB), and registered in www.clinicaltrials.gov (identifier: NCT03733158).

### Study participants

Following IRB approval and written informed consent, 32 experienced anesthesiologists with at least 2 years of clinical experience participated in this study. No anesthesiologist had any prior experience with the new flexible tip bougie, but each was experienced with the standard bougie and all had performed a minimum of 500 endotracheal intubations using the Macintosh laryngoscope.

### Intubation devices

**Fig. 1:**
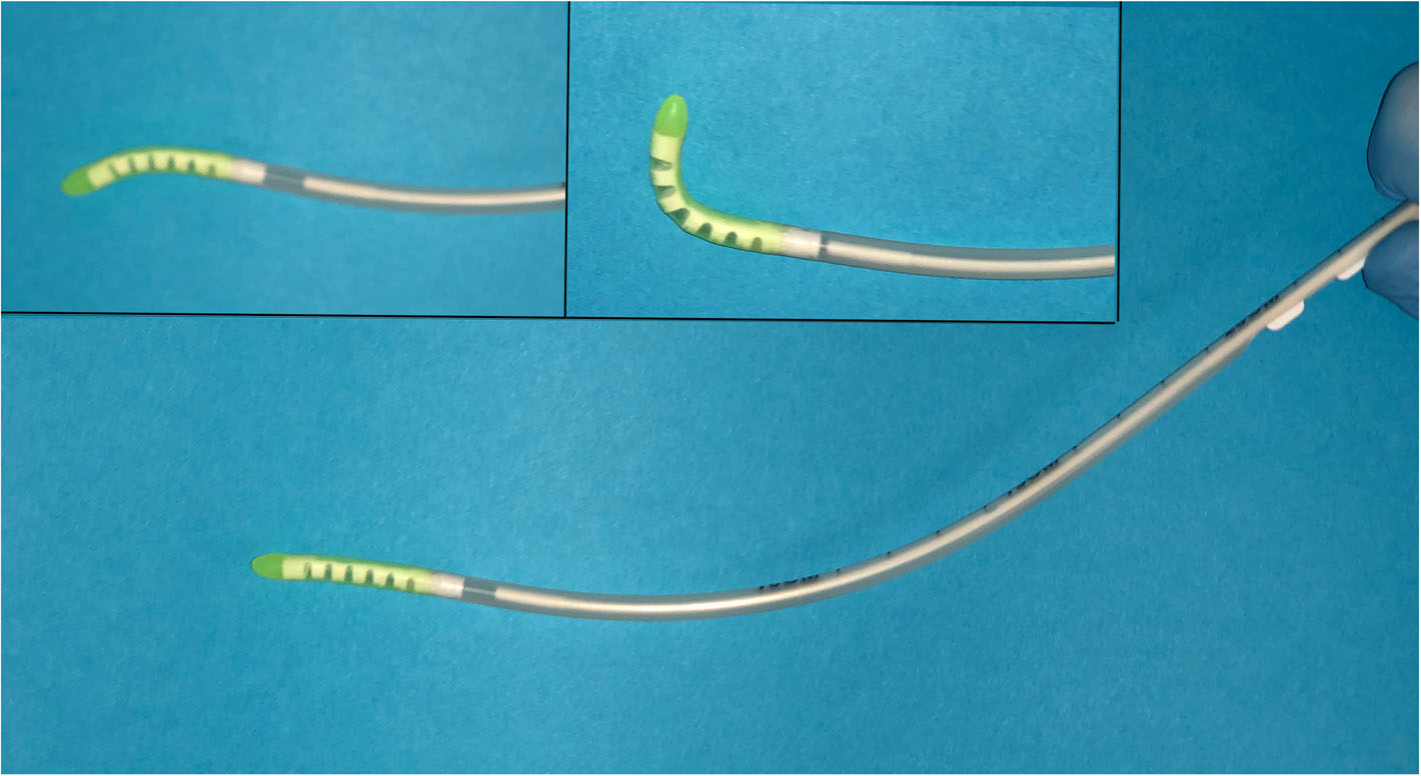
The new Flexible tip bougie catheter

All intubation procedures were performed using a Macintosh blade size 3 (Heine Optotechnik, Herrsching, Germany) and one out of two bougies:

1. The standard bougie for difficult intubation (Sumi, Sulejówek, Poland);
2. The new flexible tip bougie (FMDSS Construct Medical, Hawthorn, Austria, Fig. 1).

Tracheal tubes (Portex, St. Paul, MN, USA) with an internal diameter of 7.5 mm were used for all intubations. Before each intubation attempt, the endotracheal tube and the manikin’s airway were thoroughly lubricated using an airway lubricant for training manikins (Laerdal, Stavanger, Norway). A regular 20 cc syringe (B. Braun Melsungen AG, Hessen, Germany) was used for cuff inflation.

### Study protocol

Each anesthesiologist participated a standardized 5 min lasting practical demonstration of the flexible tip bougie and the standard bougie by one of the investigators. Once completed, each anesthesiologist performed tracheal intubation with both devices in a Laerdal Airway Management Trainer (Laerdal, Stavanger, Norway) in 2 scenarios:

1. normal airway in the supine position
2. normal airway with the neck immobilized using a hard-cervical collar.

Afterwards, anesthesiologists performed tracheal intubation in a SimMan 3G simulator (Laerdal, Stavanger, Norway) in 6 different airway scenarios:

A) Normal airway;
B) Tongue edema;
C) Pharyngeal obstruction;
D) Manual cervical inline stabilization;
E) Cervical collar stabilization;
F) Cervical collar stabilization and pharyngeal obstruction.

Once anesthesiologists completed all intubations in all eight scenarios, they were asked to perform another endotracheal intubations on the Laerdal Airway Management Trainer with a normal airway using both devices. The intubation procedure was closely monitored by one of the investigators, to certify, that intubations using both devices were performed in an adequate manner. If needed, endotracheal intubations were repeated until both the anesthesiologist and the investigator were satisfied.

For the study, the SimMan 3G simulator (Laerdal, Stavanger, Norway) was placed on a hard, flat table to simulate an “in the bed” scenario. Anesthesiologists were instructed to intubate the manikin with one of the two devices, insufflate the cuff of the tube, attach a bag valve mask, and provide one breath to ventilate the lungs of the simulator for an overall of six different airway scenarios:

- Normal airway;
- Tongue edema;
- Pharyngeal obstruction;
- Manual cervical inline stabilization;
- Cervical collar stabilization;
- Cervical collar stabilization and pharyngeal obstruction.

Both, the sequence of the intubation devices and the six airway scenarios were randomized using the research randomizer (randomizer.org).

### Measurements

The primary endpoint was the rate of successful placement of the tracheal tube in the trachea with a maximum of three intubation attempts. A failed intubation attempt was defined as an attempt in which the trachea was not intubated, or lasted longer than 120 s [15]..

The secondary endpoint was time required for successful tracheal intubation. The time for successful intubation, was defined as the time between insertion of the blade between the teeth until the manikin was successfully ventilated, confirmed by lung insufflation during bas-mask ventilation [15].

Number of intubation attempts, and number of optimization maneuvers required (re-adjustment of manikin’s head position, and BURP -backward, upward, and rightward pressure to the larynx- maneuver performed by a researcher), served as additional secondary endpoints. All outcomes were assessed by one of the researchers. A researcher further scored the severity of dental compressions, which was assessed by the number of audible teeth clicks (0; 1; ≥2) with the Laerdal airway trainer, and by a grading of pressure of the teeth (none = 0; mild = 1; moderate/serve ≥2) on the SimMan 3G simulator. At the end of each scenario, each participant scored the ease of use of each intubation device on a visual analogue scale ranging from 0 (extremely easy) to 100 (extremely difficult).

### Sample size

The sample size was calculated with the G*Power 3.1 software, and the two-tailed t test was applied (Cohen’s d, 0.8; alpha error, 0.05; power, 0.95). We calculated that at least 28 participants would be required (paired, 2-sided). To minimalize the impact of potentially data loss, we planned to enroll up to 32 anesthesiologists into this study.

### Statistics

All statistical analyses were performed with statistical package STATISTICA 13.3EN (TIBCO Inc., Tulsa, OK, USA). The normal distribution of data was tested using the Kolmogorov-Smirnov test. Results obtained from each trial were compared using two-way repeated-measurements analysis of variance for intubation time. Fisher’s exact test was used for the success rate. The participants’ subjective opinions were compared with the use of the Stuart-Maxwell test. Data were presented as medians and interquartile range (IQR) or number and percentage (%). The α-error level for all analyses was set as *P* < .05.

## Results

Between January 2019 and May 2019, a total of thirty-two anesthesiologists were recruited. The median clinical experience of the anesthesiologists was 3.5 years (Inter Quartile Range IQR; 2.5–5). Each anesthesiologist had previously performed at least 500 endotracheal intubations using the Macintosh laryngoscope, and none had any experience with the new flexible tip bougie, but with the standard bougie.

### Scenario 1: Normal airway

All anesthesiologists successfully intubated the trachea with the first intubation attempt using both bougies (Table 1).

**Table 1:** Data from intubation in Scenario A: Normal airway. Data are presented as median (IQR), or as number (percentage)

parameter	standard bougie	flexible tip bougie catheter	***p***-value
Overall success rate, %	32 (100%)	32 (100%)	NS
Duration of 1st intubation attempt, sec	27 (21.5–36)	25 (19–34)	NS
Number of intubation attempts (%)			
1	32 (100%)	32 (100%)	
2	0 (0%)	0 (0%)	
3	0 (0%)	0 (0%)	
Median (IQR)	1 (1–1)	1 (1–1)	NS
Number of optimization maneuvers (%)			
0	30 (94%)	31 (97%)	
1	2 (6%)	1 (3%)	
2	0 (0%)	0 (0%)	
Median (IQR)	0 (0–0)	0 (0–0)	NS
Severity of dental compression (%)			
0	14 (44%)	17 (53%)	
1	16 (50.0%)	15 (47%)	
2	2 (6%)	0 (0%)	
Median (IQR)	1 (0–1)	0 (0–1)	NS
Ease of use (1–100)	18 (10–21)	18 (10–19)	NS

*NS* Not statistically significant

### Scenario 2: tongue edema

Overall intubation success rate was 100% for both intubation devices. Successful intubation with the first intubation attempt was 22% with the bougie and 34% for the flexible tip bougie (Table 2). Use of the new flexible tip bougie was associated with less optimization maneuvers and less dental compression compared to the standard bougie.

**Table 2:** Data from intubation in Scenario B: Tongue edema. Data are presented as median (IQR), or as number (percentage)

parameter	standard bougie	flexible tip bougie catheter	***p***-value
Overall success rate, %	32 (100%)	32 (100%)	NS
Duration of 1st intubation attempt, s	44 (35–73)	40 (30–55)	**0.046**
Number of intubation attempts (%)			
1	7 (22%)	11 (34%)	
2	17 (53%)	19 (59%)	
3	8 (25%)	2 (6%)	
Median (IQR)	2 (2–2.3)	2 (1–2)	NS
Number of optimization maneuvers (%)			
0	0 (0%)	6 (19%)	
1	7 (22%)	23 (72%)	
2	25 (78%)	3 (9%)	
Median (IQR)	2 (2–2)	1 (1–1)	**< 0.001**
Severity of dental compression (%)			
0	2 (6%)	4 (13%)	
1	1 (3%)	10 (31%)	
2	29 (91%)	18 (56%)	
Median (IQR)	2 (2–2)	2 (1–2)	**0.024**
Ease of use (1–100)	56 (41–67)	45 (40–57)	**0.038**

*NS* Not statistically significant

### Scenario 3: pharyngeal obstruction

Anesthesiologists successfully intubated with the first intubation attempt with both bougies (Table 3). The use of new flexible tip bougie again caused less optimization maneuvers and less dental compression compared to the standard bougie.

**Table 3:** Data from intubation in Scenario C: Pharyngeal obstruction. Data are presented as median (IQR), or as number (percentage)

parameter	standard bougie	flexible tip bougie catheter	***p***-value
Overall success rate, %	32 (100%)	32 (100%)	NS
Duration of 1st intubation attempt, s	29 (23.5–36)	24 (20.5–32)	**0.010**
Number of intubation attempts (%)			
1	32 (100%)	32 (100%)	
2	0 (0%)	0 (0%)	
3	0 (0%)	0 (0%)	
Median (IQR)	1 (1–1)	1 (1–1)	NS
Number of optimization maneuvers (%)			
0	19 (59%)	30 (94%)	
1	13 (41%)	2 (6%)	
2	0 (0%)	0 (0%)	
Median (IQR)	0 (0–1)	0 (0–0)	**0.018**
Severity of dental compression (%)			
0	7 (22%)	15 (47%)	
1	9 (28%)	13 (41%)	
2	16 (50%)	4 (12%)	
Median (IQR)	1.5 (1–2)	1 (0–1)	**0.004**
Ease of use (1–100)	34 (22–41)	32 (20–39)	NS

*NS* Not statistically significant

### Scenario 4: manual inline stabilization

Overall rate of successful was 100% in both devices (Table 4). Successful intubation with the first intubation attempt was 94% with the flexible tip bougie compared to 59% with the standard bougie (statistically not significant). The rate of optimization maneuvers and dental compression was less if used the flexible tip bougie compared to the standard bougie.

**Table 4:** Data from intubation in Scenario D: Manual cervical inline stabilization. Data are presented as median (IQR), or as number (percentage)

parameter	standard bougie	flexible tip bougie catheter	***p***-value
Overall success rate, %	32 (100%)	32 (100%)	NS
Duration of 1st intubation attempt, s	34 (30–48)	29 (25–34)	**0.001**
Number of intubation attempts (%)			
1	27 (84%)	30 (94%)	
2	5 (16%)	2 (6%)	
3	0 (0%)	0 (0%)	
Median (IQR)	1 (1–1)	1 (1–1)	NS
Number of optimization maneuvers (%)			
0	10 (31%)	12 (37%)	
1	15 (47%)	19 (59%)	
2	7 (22%)	1 (3%)	
Median (IQR)	1 (0–1)	1 (0–1)	NS
Severity of dental compression (%)			
0	0 (0%)	6 (19%)	
1	12 (37%)	15 (47%)	
2	20 (62%)	11 (34%)	
Median (IQR)	2 (1–2)	1 (1–2)	**0.015**
Ease of use (1–100)	61 (40–72)	53 (38–69)	**0.013**

*NS* Not statistically significant

### Scenario 5: cervical collar stabilization

Overall success rate was 100% with both bougies. First intubation attempt success rate was 81% for the standard bougie and 94% for the new flexible tip bougie (Table 5). Time to intubation was shorter with the new flexible tip bougie (37 s) compared to the standard bougie (46 s, *p* = < 0.001).

**Table 5:** Data from intubation in Scenario E: Cervical collar stabilization. Data are presented as median (IQR), or as number (percentage)

parameter	standard bougie	flexible tip bougie catheter	***p***-value
Overall success rate, %	32 (100%)	32 (100%)	NS
Duration of 1st intubation attempt, s	46 (38–53)	37 (31.5–46)	**< 0.001**
Number of intubation attempts (%)			
1	26 (81%)	30 (94%)	
2	5 (16%)	1 (3%)	
3	1 (3%)	1 (3%)	
Median (IQR)	1 (1–1)	1 (1–1)	NS
Number of optimization maneuvers (%)			
0	9 (28%)	12 (37%)	
1	15 (47%)	17 (53%)	
2	8 (25%)	3 (9%)	
Median (IQR)	1 (0–1.3)	1 (0–1)	NS
Severity of dental compression (%)			
0	0 (0%)	4 (12%)	
1	9 (28%)	13 (41%)	
2	23 (72%)	15 (47%)	
Median (IQR)	2 (1–2)	1 (1–2)	NS
Ease of use (1–100)	72 (53–79)	60 (45–71)	**0.014**

*NS* Not statistically significant

### Scenario 6: cervical collar stabilization and pharyngeal obstruction

Overall success rate (100% vs. 94%, not significant) as well as first attempt success rate (72% vs 66%, not significant) was higher with the new flexible tip bougie compared to the standard bougie (Table 6). The new flexible tip bougie again caused less optimization maneuvers (*p* = < 0.001) and less dental compression (*p* = 0.008) compared to the standard bougie.

**Table 6:** Data from intubation in Scenario F: Cervical collar stabilization and pharyngeal obstruction. Data are presented as median (IQR), or as number (percentage)

parameter	standard bougie	flexible tip bougie catheter	***p***-value
Overall success rate, %	30 (94%)	32 (100%)	NS
Duration of 1st intubation attempt, s	53 (44–73)	44 (35–59)	**0.002**
Number of intubation attempts (%)			
1	21 (66%)	24 (72%)	
2	10 (31%)	6 (19%)	
3	1 (3%)	3 (9%)	
Median (IQR)	1 (1–2)	1 (1–1.3)	NS
Number of optimization maneuvers (%)			
0	3 (9%)	10 (31%)	
1	8 (25%)	19 (59%)	
2	21 (66%)	3 (9%)	
Median (IQR)	2 (1–2)	1 (0–1)	**< 0.001**
Severity of dental compression (%)			
0	0 (0%)	7 (22%)	
1	6 (19%)	10 (31%)	
2	26 (81%)	15 (47%)	
Median (IQR)	2 (2–2)	1 (1–2)	**0.008**
Ease of use (1–100)	83 (72–90)	69 (54–77)	**< 0.001**

*NS* Not statistically significant

The new flexible tip bougie was assessed by the participating anesthesiologists to be easier to use in all difficult, but not in the normal airway scenario.

## Discussion

The purpose of this manikin study was to compare the flexible tip bougie with the standard bougie as aids for endotracheal intubation, using simulated normal and difficult airway scenarios. During normal simulated airways scenarios, overall and first attempt success rates, number of intubation attempts, number of optimizing maneuvers and complications such as dental compression, and ease of use were similar for the flexible tip bougie and the standard bougie. This might be mostly based on the fact, that participating anesthesiologists were previously familiar with the standard bougie. This is also reassuring, that the new flexible tip bougie did not require additional previous extensive training to familiarize with the slightly different technique.

Generally, bougies are advocated to facilitate intubations, when external manipulation seemed to improve glottic visualization [14]. The prime advantage of flexible tip bougie — ability to negotiate hyper acute curves — was therefore further tested by creating a simulated scenario of difficult intubation. Flexible tip bougie was able to achieve comparable overall success rate with reduced number of intubation attempts and optimization maneuver. We further investigated the two different bougie’s in predicted difficult intubation scenarios such as cervical spine immobilization. Importantly, we observed a trend whereby the use flexible tip bougie appears to be superior to standard bougie with comparable success rates, reduced number of intubation attempts and time to endotracheal intubation. Advantages of decreased cervical movements and high first-time success rate of tracheal intubation have been described previously [16]. The application of manual in-line stabilization and cervical collar are known to worsen glottic visualization by at least one grade – thereby significantly impede intubation further leading to difficult laryngoscopy, increased hypoxia times and poor outcomes [11, 17]. Finally, a more complex scenario was created where we combined the cervical collar stabilization and pharyngeal obstruction together, and found improved overall success rate with the flexible tip bougie, earlier intubation by 9 s with number of optimization attempts restricted to 0–1 in the majority. The reduced time to intubation in cervical immobilization scenarios indicate that navigation with the flexible tip bougie is less time consuming compared to the standard bougie.

A recent study in the emergency room setting compared the standard bougie with an endotracheal tube equipped with a stylet and reported, that using a bougie resulted in higher first attempt intubation success rate and similar time to intubation (36 vs. 38 s, not significant) [8]. Another comparative manikin study evaluated the standard bougie and a fiberoptic stylet in difficult airways scenario and reported comparable mean time to successful intubation (31 vs 45 s, not significant) [18]. Previous studies further reported increased first pass success rate by standard bougie in simulated settings [6, 11, 19].

We noticed a decreased rate of dental compressions with the flexible tip bougie in difficult scenarios, except cervical collar scenario. Previous work suggests that the strain is not affected by the level of experience or training or number of previous intubations, however it varies widely across intubators and the severity may be reduced by the application of alcohol protective pads [20]. In our study, reduced strain may be attributed due to improved maneuverability of flexible tip bougie.

Standard bougies are commonly used as a rescue device for unexpected difficult intubations, most likely due to poor glottic visualization. Maneuvers such as “rotations” – signs like “clicks” and “hold up” are considered assurances of tracheal intubation [21, 22]. In such scenarios, the maneuverability of the flexible tip bougie can be utilized in conjunction with video laryngoscopes, to finally achieve endotracheal intubation— under indirect visualization [23, 24]. Although further research is needed with the flexible tip bougie, we expect that the utilization of flexible tip bougie with video laryngoscope may be helpful in difficult airways situations. Additionally, flexible tip bougie can be manipulated to rotate with a one-handed integrated slider, however excessive rotational force and additional help from a bystander is needed to achieve free rotation with standard bougie [25].

Our analysis should be interpreted with several limitations. It is worth noting that our study is a preliminary manikin study, the results of which are often times difficult to extrapolate to humans. Time to perform intubation is usually quicker in simulated models and the manikin does not fully reproduce laryngoscopic conditions in real patients. Anyhow, a reduction of a few seconds in any manner doesn’t seem to be clinically relevant. Although not investigated in this study, the endotracheal tube may encounter resistance when railroaded over the bougie, and therefore makes intubation over the bougie more difficult [26, 27]. Airway perforation and soft tissue damage are important clinical concerns, although there is limited published evidence to support [28]. Based on the nature of this research, it was impossible to blind neither the intubators nor the assessing researchers. We included only experienced anesthesiologists which may be partly responsible for the high success rates, and faster time to intubation. However, results of this study are difficult to generalize to physicians with variable level of experience. We also did not standardize the techniques for using the bougies. There might be a small variety of techniques used in this study, which is mostly due to the fact, that all anesthesiologists had previous clinical experience with the standard bougie. Interestingly, although not having any previous experience with the flexible tip bougie, anesthesiologists achieved a high success rate of intubation, indicating a fast learning curve with the new device. However, this needs to be proven in less experienced providers. Finally, intubation using a bougie is considered a rescue technique for unexpected difficult intubations. Although also investigated in this manikin study, routine use of bougies in expected difficult intubations is currently not recommended.

## CONLUSIONS

The newly introduced flexible tip bougie offered similar overall and first attempt success rates in normal airway scenarios compared to the standard bougie. In more difficult airway scenarios, the flexible tip bougie was associated with similar overall success rates, but less intubation attempts, less adjustment maneuvers, less dental compression, and assessment of easier to use compared to standard bougie. It appears that the innovative flexible tip bougie might a valuable airway adjunct for difficult intubations. Further research in the human clinical setting is indicated to confirm these findings and possibly address the limitations of this study.

## Data Availability

The datasets used and/or analyzed during the current study available from the corresponding author on request.

## Abbreviations

IRB: Institutional Review Board
NCT: National Clinical Trial number
BURP: Backward, upward, and rightward pressure to the larynx- maneuver
IQR: Inter Quartile Range
